# Generalized $\mathbb {XOR}$ Operation and the Categorical Equivalence of the Abbott Algebras and Quantum Logics

**DOI:** 10.1007/s10773-023-05355-3

**Published:** 2023-05-04

**Authors:** Dominika Burešová

**Affiliations:** Czech Technical University in Prague, Department of Cybernetics, Faculty of Electrical Engineering, Prague, Czech Republic

**Keywords:** Boolean algebra, Abbott orthoimplication algebra, Orthomodular lattice, Symmetric difference, Categorical equivalence

## Abstract

Considering the inference rules in generalized logics, J.C. Abbott arrives to the notion of orthoimplication algebra (see Abbott (1970) and Abbott (Stud. Logica. 2:173–177, XXXV)). We show that when one enriches the Abbott orthoimplication algebra with a falsity symbol and a natural $\mathbb {XOR}$-type operation, one obtains an orthomodular difference lattice as an enriched quantum logic (see Matoušek (Algebra Univers. 60:185–215, 2009)). Moreover, we find that these two structures endowed with the natural morphisms are categorically equivalent. We also show how one can introduce the notion of a state in the Abbott $\mathbb {XOR}$ algebras strenghtening thus the relevance of these algebras to quantum theories.

## Introduction and Basic Notions

Recently there has been an effort to soundly introduce and study the notion of a symmetric difference in orthomodular lattices and posets (see  [3, 4, 8, 9, 12], etc.). In the relation to quantum axiomatics, the idea has been to enrich the “quantum logics” with a kind of a $\mathbb {XOR}$ operation. There are several non-Boolean orthomodular lattices that allow for this operation and become thus “nearly Boolean”.

In this note, we introduce a $\mathbb {XOR}$ operation by extending the language of the Abbott implication algebras. The technical side overlaps to a certain extent with a synthesis of [1, 2], the presence of the falsity symbol 0 and the $\mathbb {XOR}$ operation makes a nead for a modified formulation in places. Also, we provide a direct proof of the journey “from orthomodular lattices to Abbott algebras” making thus the envisaged equivalence more insightful. At that, as a by-product, this introduces an “Abbott operation” in the class of orthomodular lattices and may allow for another algebraic investigation (see also [6]).

Thought we consider the algebras endowed with a $\mathbb {XOR}$ operation, it may be noted that we also provide another proof of the equivalence of the Abbott algebras with the orthomodular lattices (forgetting the operation $\mathbb {XOR}$). As commented at the end of this paper, this allows us to translate all algebraic and state space features of the orthomodular lattices into the Abbott algebras.

For a last introductory remark, recall that the Abbott algebras are originally mostly studied without the falsity nullary element. It should be noted that we could also introduce “a partial $\mathbb {XOR}$ operation” into the algebras. But a potential interpretation of such a notion does not seem naturally possible in the quantum logic theory.

Let us take up the subject proper. Our basic definition reads as follows.

### Definition 1.1

Let $(A,~0,~\cdot ,~\Delta )$ be an algebra with a nullary operation 0 and two binary operations $\cdot$ and $\Delta$. Let the operations fulfill the following requirements (we omit the symbol $\cdot$ writing simply *ab* instead of $a \cdot b$; let $a,b,c \in A$).

1. $$(ab)a = a$$,
2. $$(ab)b = (ba)a$$,
3. $$a \bigl ((ba)c \bigr ) = ac$$,
4. $$0a = bb$$,
5. $$(a \Delta b) \Delta c = a \Delta (b \Delta c)$$,
6. $$a \Delta bb = a0$$,
7. $$bb \Delta a = a0$$,
8. $$(a \Delta b) \bigl ( (ab)b \bigr ) = aa$$.

Then $(A,~0,~\cdot ,~\Delta )$ is said to be **the Abbott** $\mathbb {XOR}$ **algebra**.

## Results

Before we pass to our results, let us recall a few properties of the operation $\cdot$ of the algebras studied.

### Proposition 2.1

Let $(A,~0,~\cdot ,~\Delta )$ be the Abbott $\mathbb {XOR}$ algebra. Then the following statements hold true $(a, b, c \in A)$:

(i) $$aa = 1$$,
(ii) $$1a = a$$,
(iii) $$a1 = 1$$,
(iv) $$ab = ba \implies a = b$$,
(v) $$a(ba) = 1$$,
(vi) $$ab = 1 \implies a(bc) = ac$$,
(vii) $$ab = 1 \implies (ba)(ac) = 1$$.

### Proof

The proof of the *Proposition 1.2* can be obtained as an interplay of the results of [1] and [2]. Since the calculus in the Abbott algebras is rather non-standard and since we want to preserve the self-containedness, let us very briefly recall the proofs. One uses the adequate axioms of *Definition 1.1*. *Ad (i):* $a(ab) = \bigl ( (ab)a \bigr ) (ab) = ab$, so $\bigl ( (ab)a \bigr ) a = \bigl ( a(ab) \bigr ) (ab) = (ab)(ab)$ and therefore $(aa) = (ab)(ab) = \bigl ( (ab)b \bigr ) \bigl ( (ab)b \bigr ) = \bigl ( (ba)a \bigr ) \bigl ( (ba)a \bigr ) = (ba)(ba) = bb$. *Ad (ii):* $1a = (aa)a = a$. *Ad (iii):* $a1 = a(aa) = aa = 1$. *Ad (iv):* $ab = ba \implies a = (ab)a = (ba)a = (ab)b = b$. *Ad (v):* $a(ba) = a \bigl ( (ba)(ba) \bigr ) = a1 = 1$. *Ad (vi):* $ab = 1 \implies a(bc) = a \bigl ( (1b)c \bigr ) = a \Bigl ( \bigl ( (ab)b \bigr ) c\Bigr ) = a \Bigl ( \bigl ( (ba)a \bigr ) c \Bigr ) = ac$. *Ad (vii):* $ab = 1 \implies (bc)(ac) = bc \bigl ( a(bc) \bigr ) = 1$. $\square$

Let us now introduce another algebraic structure (see [5]-the structure of orthomodular lattices alias quantum logics). As known, the orthomodular lattices found its application in quantum theories. We enrich them with another operation, $\Delta$.

### Definition 2.2

Let us consider a 7-tuple $(D,~0,~1,~\wedge ,~\vee ,~^{\perp },~\Delta )$ where $(D,~0,~1,~\wedge ,~\vee ,~^{\perp })$ is an orthomodular lattice, and the binary operation $\Delta$ has the following properties $(a,b \in D)$:

1. the operation $\Delta$ is associative,
2. $$1 \Delta a = a^{\perp }, a \Delta 1 = a^{\perp }$$,
3. $$a \Delta b \le a \vee b$$.

Then $(D,~0,~1,~\wedge ,~\vee ,~^{\perp })$ is said to be **the orthomodular difference lattice** (see [9]).

### Theorem 2.3

Let $\mathcal {A}$ be the category of Abbott $\mathbb {XOR}$ algebras with the corresponding (universal algebra) morphisms, and let $\mathcal {D}$ be the category of orthomodular difference lattices with the corresponding (universal algebra) morphisms. Then the categories $\mathcal {A}$ and $\mathcal {D}$ are equivalent.

### Proof

Let $A \in \mathcal {A}$ and let us see how we can view *A* as an object of $\mathcal {D}$. Let us first endow *A* with a partial ordering. Let us introduce the partial ordering in *A* by requiring $a \le b$ if $ab = 1$. Then $0 \le a \le 1$ for all $a \in A$ because $0a = 1$ and $a1 = 1$. Let us show that $\le$ is a partial ordering with a least (resp. greatest) elements 0 (resp. 1). Indeed, $a \le a$ since $aa = 1$, and if $a \le b$ and $b \le a$, then $ab = 1 = ba$ and therefore $a = b$. Further, if $a \le b$ and $b \le c$ then $ab = 1$ and $bc = 1$. It follows from *Proposition 1.2 (vii)* that $(ba)(ac) = 1$. Then $bc \le ac$ but $bc = 1$ and therefore $1 \le ac$. So $ac = 1$ and therefore $a \le c$.

Let us see that *A* is a lattice with respect to $\le$. We claim that $a \vee b = (ab)b$. To see that, we have $a\bigl ( (ab)b \bigr ) = a \bigl ( (ba)a \bigr ) = 1$ and therefore $a \le (ab)b$ which means that $a \le a \vee b$. Analogously, $b \le a \vee b$. Moreover, if $a \le c$ and $b \le c$ then $ac = 1$ and $bc = 1$. Considering $ac=1$ (and correcting [1]), we infer that $(cb)(ab) = 1$ (Proposition 1.2, (*vii*)). This implies that $\bigl ( (ab)b \bigr ) \bigl ( (cb)b \bigr ) = 1$ and therefore $(ab)b \le (cb)b$. So $a \vee b = (ab)b \le (cb)b = (bc)c = 1c = c$ and hence $a \vee b \le c$. This shows that *A* is a lattice.

With the intention to restructure *A* to make it an orthocomplemented lattice, let us set $a^{\perp } = (a0)$. We are to verify that $(a^{\perp })^{\perp } = a, a \le b \implies b \le a$ and that both equalities $a \vee a^{\perp } = 1, a \wedge a^{\perp } = 0$ are valid. Obviously, $(a^{\perp })^{\perp } = (a0)0 = a \vee 0 = a$. Further, if $a \le b$ then $b^{\perp } = (b0) \le (a0) = a^{\perp }$. Let us also see that $a \vee a^{\perp } = 1$ and $a \wedge a^{\perp } = 0$. We have $a \vee a^{\perp } = a^{\perp } \vee a = \bigl (a0)a \bigr ) a = aa = 1$. As regards the condition on the infimum, one uses the de Morgan law to obtain $a \wedge a^{\perp } = a^{\perp } \wedge a = (a \vee a^{\perp })^{\perp } = (a \vee a^{\perp })0 = \Bigl ( \bigl ( a(a0) \bigr ) (a0) \Bigr ) 0 = (a \vee a^{\perp })0 = (10) = 0$.

It remains to verify the orthomodular law. Suppose that $a \le b$. So we have $(b0) \le (a0)$ and we see (*by Definition 1.1, 3.*) that $b = b \vee 0 = \bigl ( (b0)0 \bigr ) = (b0) \bigl ( (a0)0 \bigr ) = (b0) (a \vee 0) = (b0)a$. Since, $a(ba) = 1$ by *Proposition 2.1 (v)*, then $a \le (ba)$ and therefore we have $(ba) = \bigl ( (ba)0 \bigr ) a$. In order to verify the orthomodular law, we are to prove that $b = a \vee (a^{\perp } \wedge b)$. Let us consider the right-hand side of this equality. We obtain $a \vee (a^{\perp } \wedge b) = a \vee \bigl ( (a0) \wedge b \bigr ) = a \vee \Bigl ( \bigl (a \vee (b0) \bigr ) 0 \Bigr )=a \vee \Bigl ( \bigl ((b0) \vee a \bigr ) 0 \Bigr )=\Bigl ( a \vee \bigl ( (b0)a \bigr ) a \Bigr ) 0 = a \vee \bigl ( (ba)0 \bigr ) = \Bigl ( \bigl ((ba)0 \bigr ) a \Bigr ) a = (ba)a = b \vee a = b$.

Finally, let us check the conditions of the operation $\Delta$. The operation $\Delta$ is associative by definition. Further, $a \Delta (bb) = a \Delta 1 = (a0) = x^{\perp }$ and $(bb) \Delta a = (1 \Delta a) = (a0) = a^{\perp }$. To end up the verification, we use $(ab)b = a \vee b$ and we obtain $aa = 1 = (a \Delta b) \bigl ( (ab) b \bigr ) = (a \Delta b) \vee (a \vee b)$. Therefore we infer that $a \Delta b \le a \vee b$.

In the considerations above, we have defined an assignement $F: \mathcal {A} \rightarrow \mathcal {D}$ as a potential functor on the objects of $\mathcal {A}$ (the assignement *F* preserves the underlying set). Let us see that this assignement is functorial. Suppose that $f: A \rightarrow B$ is a morphism in $\mathcal {A}$. So $f(ab) = f(a)f(b)$, $f(0) = 0$ and $f(a \Delta b) = f(a) \Delta f(b)$. We have to check that *f* is a morphism in $\mathcal {D}$. For that, suppose that $a \vee b = c$ in $\mathcal {A}$. So it means that $c = (ab)b$. Thus $f(c) = \bigl ( f(a)f(b) \bigr ) f(b)$. This implies that $f(c) = f(a) \vee f(b)$. Further, we have to check that $f(a^{\perp }) = f(a)^{\perp }$. But $a^{\perp } = (a0)$ and therefore $f(a^{\perp }) = f(a0) = f(a)f(0) = f(a)0$, and hence $f(a)^{\perp } = f(a^{\perp })$. Thus we have checked that *F* is indeed a functor from $\mathcal {A}$ to $\mathcal {D}$.

We shall now construct a functor, *G*, $G : \mathcal {D} \rightarrow \mathcal {A}$. Let us take $D \in \mathcal {D}$. Then *G*(*D*) remains with the same underlying set. We define the object *G*(*D*) as follows: If $a \in G(D)$ and $b \in G(D)$, then $ab = (a \vee b)^{\perp } \vee b$, and $a \Delta b$ is copied from *D*. Let us check that *G*(*D*) sends a morphism of $\mathcal {D}$ into a morphism of $\mathcal {A}$. We first have to check that the axioms of *G*(*D*) make it an Abbott $\mathbb {XOR}$ algebra.

1. $$(ab)a = a$$; we have $$\Bigl ( \bigl ( (a \vee b)^{\perp } \vee b \bigr ) \vee a \Bigr )^{\perp } \vee a = \bigl ( (a \vee b) \wedge b^{\perp } \wedge a^{\perp } \bigr ) \vee a = \bigl ( (a \vee b) \wedge (a \vee b)^{\perp } \bigr ) \vee a = 0 \vee a = a$$.
2. $(ab)b = (ba)a$; we have $(ab)b = \bigl ( (a \vee b)^{\perp } \vee b \bigr ) ^{\perp } \vee b = \bigl ( (a \vee b) \wedge b^{\perp } \bigr ) \vee b$. Since the triple $b, b^{\perp }, a \vee b$ is compatible in *D*, we can use distributivity (see e.g. [5]). Hence, the latter formula gives us $(a \vee b \vee b) \wedge (b^{\perp } \vee b) = a \vee b$. Analogously, $\bigl ( (b \vee a)^{\perp } \vee a \bigr ) ^{\perp } \vee a = b \vee a$ and so the equality is valid.
3. $a \bigl ( (ba)c \bigr ) = ac$. Prior to verifying this condition, let us make a preliminary observation. Consider the elements *x* and $y^{\perp } \wedge x$. Then $x \ge y^{\perp } \wedge x$. So the orthomodular law gives us $x = (y^{\perp } \wedge x) \vee \bigl ( (y^{\perp } \wedge x)^{\perp } \wedge x \bigr ) = (y^{\perp } \wedge x) \vee \bigl ( (y \vee x^{\perp }) \wedge x \bigr )$. Let us verify the axiom proper. We have $a \bigl ( (ba)c \bigr ) = a \Bigl ( \bigl ( (b \vee a)^{\perp } \vee a \bigr ) c \Bigr ) = a \Bigl ( \bigl ( (b^{\perp } \wedge a^{\perp }) \vee a \bigl ) c \Bigr )$. For the sake of transparency, let us write $y =b^{\perp } \wedge a^{\perp }$. Hence we have $a \bigl ( (ba)c \bigr ) = a \bigl ((y \vee a) c \bigr ) = a\Bigl (\bigl ((y \vee a)\vee c \bigr )^{\perp } \vee c \Bigr ) = \biggl ( a \vee \Bigr ( \bigl ( (y \vee a)^{\perp } \bigr ) \wedge c^{\perp } \Bigr ) \vee c \biggr )^{\perp } \vee \biggl ( \Bigr ( \bigl ( (y \vee a)^{\perp } \bigr ) \wedge c^{\perp } \Bigr ) \vee c \biggr ) = \biggl ( a^{\perp } \wedge \Bigr ( \bigl ( (y \vee a)^{\perp } \wedge c^{\perp } \bigr ) \vee c \Bigr )^{\perp } \biggr ) \vee \Bigr ( \bigl ( (y \vee a)^{\perp } \wedge c^{\perp } \bigr ) \vee c \Bigr ) = \Biggl ( a^{\perp } \wedge \biggl ( \Bigr ( \bigl ( (y \vee a)^{\perp } \wedge c^{\perp } \bigr ) \vee c^{\perp } \Bigr )^{\perp } \wedge c^{\perp } \biggr ) \Biggr ) \vee \Bigl ( \bigl ( (y \vee a)^{\perp } \wedge c^{\perp } \bigr ) \vee c \Bigr ) = \Bigl ( (a^{\perp } \wedge c^{\perp }) \wedge \bigl ( (y \vee a)^{\perp } \wedge c^{\perp } \bigr )^{\perp } \Bigr ) \vee \bigl ( (y^{\perp } \wedge a^{\perp } \wedge c^{\perp }) \vee c \bigr ) = \Bigl ( (a^{\perp } \wedge c^{\perp }) \wedge (y \vee a \vee c) \vee (y^{\perp } \wedge a^{\perp } \wedge c^{\perp }) \Bigr )\vee c$. So we have
  $$\begin{aligned} a \bigl ( (ba)c \bigr ) = \biggl ( (a^{\perp } \wedge c^{\perp }) \wedge \Bigl ( \bigl ( (b^{\perp } \wedge a^{\perp }) \vee a \bigr ) \vee c) \Bigr ) \biggr ) \vee \Bigl ( \bigl ( (b^{\perp } \wedge a^{\perp })^{\perp } \wedge a^{\perp } \bigr ) \wedge c^{\perp } \Bigr ) \vee c \end{aligned}$$
  Let us set $u = (a^{\perp } \wedge c^{\perp }) \wedge \bigl ( (b^{\perp } \wedge a^{\perp }) \vee a \vee c) \bigr ) \vee \bigl ( (b^{\perp } \wedge a^{\perp })^{\perp } \wedge a^{\perp } \wedge c^{\perp } \bigr )$. Writing $x = a^{\perp } \wedge c^{\perp }$ and $y = b^{\perp } \vee a^{\perp }$, let us use the orthomodular law formula derived at the beginning of this proof. We obtain $a^{\perp } \wedge c^{\perp } = x = (y^{\perp } \vee x) \vee \bigl ( (y^{\perp } \wedge x)^{\perp } \wedge x \bigr ) = u$. As a result, we have $a \bigl ( (ba)c \bigr ) = (a^{\perp } \wedge c^{\perp }) \vee c = ac$, which we wanted to prove.
4. $$0a = bb$$; we have $$(0a) = (0 \vee a)^{\perp } \vee a = (1 \wedge a^{\perp }) \vee a = a^{\perp } \vee a = 1 = bb$$.
5. $(a \Delta b) \Delta c = a \Delta (b \Delta c)$, the operation $\Delta$ is associative in *A* as well as in the corresponding orthomodular lattice.
6. $$a \Delta bb = a0$$; we have $$(a0) = (a \vee 0)^{\perp } \vee 0 = a^{\perp } = a \Delta 1 = a \Delta bb$$.
7. $$bb \Delta a = a0$$; we have $$(a0) = (a \vee 0)^{\perp } \vee 0 = a^{\perp } = 1 \Delta a = bb \Delta a$$.
8. $(a \Delta b) \bigl ( (ab)b \bigr ) = (ab)b$; we have $(ab)b = a \vee b$ and therefore $(a \Delta b ) \vee (a \vee b) = a \vee b$. So $a \Delta b \le a \vee b$.

$$\square$$

Making use of the above equivalence of $\mathcal {A}$ and $\mathcal {D}$ we can express the notion of compatibility in the Abbott $\mathbb {XOR}$ algebras. Suppose that $a, b \in A$, $A \in \mathcal {A}$. We say that **the elements** ***a,b*** **are compatible** in *A* if they generate a Boolean subalgebra of *A*. This notion is associated with “commonsurability” in a quantum experiment (see e.g. [5]). It can be captured in the Abbott $\mathbb {XOR}$ algebras as well, though not as economically as one would hope for.

### Proposition 2.4

Let *A* be an Abbott $\mathbb {XOR}$ algebra and let $a,b \in A$. Then *a*, *b* are compatible in *A* if either of the following two conditions is satisfied:

1. $$a = \Biggl ( \biggl ( \Bigl ( \bigl ( (a0)(b0) \bigr ) (b0) \Bigr ) 0 \biggr ) \biggl ( \Bigl ( \bigl ( (a0)b \bigr ) b \Bigr )0 \biggr ) \Biggr ) \biggl ( \Bigl ( \bigl ( (a0)b \bigr ) b \Bigr )0 \biggr )$$
2. $$a \Delta b = \Biggl ( \biggl ( \Bigl ( \bigl ( (a0)b \bigr ) \Bigr ) 0 \biggr ) \biggl ( \Bigl ( \bigl ( a(b0) \bigr ) (b0) \Bigr ) 0 \biggr ) \Biggr ) \biggl ( \Bigl ( \bigl ( a(b0) \bigr ) (b0) \Bigr ) 0 \biggr )$$

A corollary: *A* is a Boolean algebra if and only if either of the above equalities is valid for any $a,b \in A$.

### Proof

If we rewrite the equality *1.* in the corresponding orthomodular lattice, we obtain $a = (a^{\perp } \vee b^{\perp })^{\perp } \vee (a^{\perp } \vee b)^{\perp } = (a \wedge b) \vee (a \wedge b^{\perp })$. Analogously, we can derive that $a \Delta b = (a \wedge b^{\perp }) \vee (b \wedge a^{\perp })$. Either of the above formulas for *a* and $a \Delta b$ guarantee that *a*, *b* are compatible (see e.g. [9]). $\square$

It may be noted that if one is allowed to use 3 variables, *A* is Boolean if and only if $a(bc) = (ab)(ac)$ (see [1]). A two variable formula for *A* to be Boolean can also be derived from this 3 variable formula.

Let us illustrate Theorem 2.3 on one example.

### Example 2.5

Let $P = \{1, 2, 3, 4\}$ and let us consider the orthomodular difference lattice $(L, 0, 1, \vee , \wedge , ^{\perp }, \Delta )$, where *L* is the collection of all subsets of *P* of an even cardinality, 0 is the empty set, $1 = P$, the operation $^{\perp }$ is the complementation operation and $\Delta$ is the symmetric difference in *P*. By the previous theorem, this orthomodular lattice could be viewed as an Abbott $\mathbb {XOR}$ algebra when we set $(AB) = (A \vee B)^{\perp } \vee B$. The Cartesian product of *L* interpreted in the corresponding categories presents a prominent example in the quantum logic theory. It is a merely matter of taste which algebraic language we adopt to it, the technicalities may seem equally complex.

For a potential application within the quantum logic theory, let us introduce the notion of a state.

### Definition 2.6

Let *A* be an Abbott $\mathbb {XOR}$ algebra. Let $s: A \rightarrow [0,1]$ be a mapping that satisfies the following conditions $(a,b,c \in A)$:

1. $$s(aa) = 1$$,
2. if $$a(b0) = bb$$, then $$s \bigl ( (ab)b \bigr ) = s(a) + s(b)$$,
3. $$s(a \Delta b) \le s(a) + s(b)$$.

Then *s* is said to be **a state in** *A* .

### Proposition 2.7

Let $\mathcal {A}$ be equivalent to $\mathcal {D}$ in the sense of Theorem 1.4. If $A \in \mathcal {A}$ and *s* is a state of *A* then *s* can be viewed as an “orthomodular” state of *F*(*A*), and vice versa.

### Proof

Recall ([4]) that a state on $\mathcal {D}$ is defined as follows $(a,b \in \mathcal {D})$:

1. $$s(1) = 1$$
2. If $$a \le b^{\perp } \implies s(a \vee b) = s(a) + s(b)$$
3. $$a (a \Delta b) \le s(a) + s(b)$$.

It is easy to see that the state space of *A* is isomorphic (via the isomorphism of *Theorem 1.4*) with the state space of $D = F(A)$. $\square$

Let us summarize main results of our paper. The category $\mathcal {A}$ of the Abbott $\mathbb {XOR}$ algebras is equivalent to the category $\mathcal {D}$ of the orthomodular difference lattices, and the respective state spaces are isomorphic. So the knowledge we have acquired on $\mathcal {D}$ and on its state space can be translated into the corresponding category $\mathcal {A}$. For instance, since we know the characterization of the set-representable objects of $\mathcal {D}$, and these are precisely those that have an “abundance” of two-valued states (see  [9]), we easily derive the set-representability characterization of the Abbott $\mathbb {XOR}$ algebras. In a similar vein, we can find Abbott $\mathbb {XOR}$ algebras without any state or with a precisely one state (see [7] and [12]). Also, we find that the free Abbott $\mathbb {XOR}$ algebra over 2 generators contains precisely 128 elements and the free algebra over 3 generators is infinite (see [10, 11], etc.). A specific line of algebraic nature is the notion of modularity in the Abbott algebras. We intend to consider this notion elsewhere.
